# The application of Large Language Models to the phenotype-based prioritization of causative genes in rare disease patients

**DOI:** 10.1038/s41598-025-99539-y

**Published:** 2025-04-29

**Authors:** Şenay Kafkas, Marwa Abdelhakim, Azza Althagafi, Sumyyah Toonsi, Malak Alghamdi, Paul N. Schofield, Robert Hoehndorf

**Affiliations:** 1 Computer, Electrical and Mathematical Sciences & Engineering Division, King Abdullah University of Science and Technology, 23955 Thuwal, Saudi Arabia; 2 SDAIA-KAUST Center of Excellence in Data Science and Artificial Intelligence, King Abdullah University of Science and Technology, 23955 Thuwal, Saudi Arabia; 3 KAUST Center of Excellence for Smart Health (KCSH), King Abdullah University of Science and Technology, 23955 Thuwal, Saudi Arabia; 4 Computer Science Department, College of Computers and Information Technology, Taif University, 26571 Taif, Saudi Arabia; 5 KAUST Center of Excellence for Generative AI, King Abdullah University of Science and Technology, 23955 Thuwal, Saudi Arabia; 6 Medical Genetic Division, Department of Pediatrics, College of Medicine, King Saud University, 11461 Riyadh, Saudi Arabia; 7 Department of Physiology, Development & Neuroscience, University of Cambridge, Cambridge, CB2 3EG UK

**Keywords:** Gene prioritization, Rare diseases, Diagnosis support, Phenotypes, Large language models, Data mining, Literature mining, Computational biology and bioinformatics

## Abstract

Computational methods for identifying gene–disease associations can use both genomic and phenotypic information to prioritize genes and variants that may be associated with genetic diseases. Phenotype-based methods commonly rely on comparing phenotypes observed in a patient with databases of genotype-to-phenotype associations using measures of semantic similarity. They are constrained by the quality and completeness of these resources as well as the quality and completeness of patient phenotype annotation. Genotype-to-phenotype associations used by these methods are largely derived from the literature and coded using phenotype ontologies. Large Language Models (LLMs) have been trained on large amounts of text and data and have shown their potential to answer complex questions across multiple domains. Here, we evaluate the effectiveness of LLMs in prioritizing disease-associated genes compared to existing bioinformatics methods. We show that LLMs can prioritize disease-associated genes as well, or better than, dedicated bioinformatics methods relying on pre-defined phenotype similarity, when gene sets range from 5 to 100 candidates. We apply our approach to a cohort of undiagnosed patients with rare diseases and show that LLMs can be used to provide diagnostic support that helps in identifying plausible candidate genes. Our results show that LLMs may offer an alternative to traditional bioinformatics methods to prioritize disease-associated genes based on disease phenotypes. They may, therefore, potentially enhance diagnostic accuracy and simplify the process for rare genetic diseases.

## Introduction

Although rare diseases individually affect a small number of people in the population, together they present a significant burden on global public health, affecting millions worldwide^1^. The diagnosis of rare diseases presents unique challenges due to their low prevalence and diverse clinical presentation in patients with the same underlying genetic lesions. Consequently, patients often endure a diagnostic odyssey, involving numerous tests and investigations over many years, before receiving a definitive diagnosis.

Most rare diseases have a genetic basis, typically involving variation in one or a few genes^2^. Next-generation sequencing (NGS) has revolutionized the diagnostic process for these diseases by enabling identification of genetic variants in individuals which are associated with the disorder. However, despite the advances in NGS approaches, achieving a molecular diagnosis remains elusive for about half of all patients^3^.

Several methods are commonly employed to reduce the number of candidate variants to consider, including the mode of inheritance, the frequency of observing the variant within different populations, and the impact a variant has on the molecular function of a gene product. However, even after these filters have been applied, there are still often tens to hundreds of candidate variants left in that individual’s genotype that need to be considered and evaluated^4^. Diverse strategies have been developed for prediction of variant pathogenicity. These methods include rule-based methods and machine learning algorithms^5^. However, accurately determining the causative variant in an individual, amidst the complexity of genetic data and phenotypic variability, remains a formidable task.

Phenotype-based methods for prioritizing genes or genotypes typically compare the phenotypes observed in a patient with the phenotypes in a genotype-to-phenotype database^6^ and rank genes or genotypes based on whether they are likely to cause the phenotypes observed in a patient. These methods rely on resources such as the Human Phenotype Ontology (HPO)^7^ and semantic similarity measures to assess the relevance of the observed phenotypes^8^. Although phenotype ontologies are manually curated and make excellent use of domain expertise, they are unavoidably incomplete and the structure reflects human decisions with consequences for the application of formal semantics^9^; genotype-to-phenotype databases are created from the literature or large-scale experiments and may be incomplete or noisy^7^; and, although a large number of semantic similarity measures have been developed, they have different biases which make them a challenge to apply consistently^10^.

In recent years, Large Language Models (LLMs) trained on large text corpora have emerged as powerful tools for natural language understanding and generation^11^. These models can potentially overcome the limitations of formal phenotype similarity-based methods by leveraging their vast knowledge and the semantic understanding implicit in their training data. Consequently, they may also be able to estimate semantic similarity as well as, or better than, ontology-dependent similarity measures. By incorporating LLMs into diagnostic workflows, we aim to improve the prioritization of disease–causing variants and enhance the genetic diagnostic yield for rare disease.

In our study, we evaluated three LLMs, GPT-3.5-turbo (model gpt-3.5-turbo-1106)^12^, GPT-4 (model gpt-4-1106-preview)^13^, and Falcon180B^14^, for ranking genes based on clinically observed phenotypes. We integrated these LLMs into diagnostic pipelines for analysing WE or WG sequencing data.

We evaluate LLMs using three synthetic datasets and one dataset of undiagnosed genetic disease patients. Through direct comparison with state-of-the-art methods, we demonstrate LLMs’ ability to enhance phenotype-based gene ranking. Moreover interactions with LLMs can provide explanations for ranking decisions and refine results, potentially serving as valuable diagnostic aids. However, we also observed several cases of “hallucinations” and other biases, highlighting the need for further refinement before considering possible application to clinical decision making. Despite these challenges, the potential of LLMs to improve rare disease diagnosis and facilitate the application of precision medicine is promising, provided that their limitations are carefully addressed.

## Materials and methods

### Datasets used

We used three benchmark datasets to conduct our experiments. The first dataset, GPCards ^15^, is a manually curated dataset of genotype–phenotype associations. We randomly selected 50 variants from distinct genes along with their corresponding clinical phenotypes from GPCards. The phenotypes in GPCards are represented as natural language terms and do not rely on a structured vocabulary or ontology. We use the GPCards dataset to develop prompts and assess the performance of the LLMs on different prompts.

The second dataset is the October 2023 release of ClinVar ^16^ a publicly accessible database detailing genomic variations and their connections to disease. We focused particularly on the new variants included in ClinVar between July 2, 2023, and October 7, 2023. From this subset of data, we randomly selected 100 variants, each from a different gene, associated with diseases in Online Mendelian Inheritance in Man (OMIM) ^17^, which are considered either “pathogenic” or “likely-pathogenic” in ClinVar. We identified the phenotypes corresponding to OMIM disease using the HPO database  ^7^ accessed on 8 October, 2023. We used time-based ClinVar data, incorporating only variants published after the LLM cut-off training date, to evaluate the potential impact of the training data on LLMs. This approach ensured that the variant-phenotype relationships in the dataset were novel and had not been seen by the LLMs or Exomiser during their training phases. ClinVar, however, has certain limitations. Phenotype information is often missing and must be inferred from OMIM identifiers, which, unlike GPCards/PAVS^18^ datasets, do not fully reflect real-world scenarios.

The third dataset is the Phenotype-Associated Variants in Saudi Arabia (PAVS) database^18^, a public database of genotype–phenotype relations identified in Saudi individuals. PAVS combines a collection of clinically validated pathogenic variants with manually curated variants specific to the Saudi population, each accompanied by its associated phenotypes mapped to HPO codes. We used the PAVS dataset to compare LLMs with ontology-based gene prioritization methods. PAVS provides direct phenotype annotations and broader variant-phenotype coverage, closely corresponding to clinical phenotype observations, unlike phenotypes in OMIM or the HPO database which collect consensus phenotypes across multiple cases. This makes PAVS advantageous over ClinVar for this specific task. We randomly selected 500 variants each from a distinct gene along with their associated phenotypes from PAVS.

For each of the benchmark sets, we generated a set of pairs (*G*, *P*) of a list of genes $G = (G_1,...,G_n)$ and a set of phenotypes $P = (P_1,...,P_m)$. The phenotypes are identical to the phenotypes from the benchmark sets (which contain genotype–phenotype relations); the list of genes *G* contains the causative gene (i.e., the genotype mapped to the underlying gene) and a set of genes randomly chosen either from all human genes or from all genes with a genotype in the benchmark set. We vary the size of the gene set *G* by randomly choosing different numbers of genes to add; the cardinality of *G* ranges from 5 to 100 (cardinalities 5, 25, 50, 75, 100). Our selection of 5 to 100 genes for the experiments aligns with findings from previous studies. A notable reduction in candidate variants is often observed after filtering variants based on pedigree or Trio data^19^. Specifically, using exomes and filtering variants by minor allele frequency (MAF), mode of inheritance, family structure, and functional impact leaves a range of 1.1 to 68.9 candidate variants to be prioritised in each patient, depending on the mode of inheritance^4^.

### Baseline methods

We evaluated several state-of-the-art methods for phenotype-based gene prioritization, all implemented in the Exomiser (version 12.1.0)^20^ system. We use three main methods as baseline: ExomeWalker^21^, PHIVE^22^, and PhenIX^23^, as well as their weighted combination (labeled “Exomiser score”). Exomiser uses phenotypes in the form of HPO terms as input and, because it is designed for ranking variants in whole exome or whole genome sequencing, it outputs a ranked list of variants from the list that the user provides. We generate a random variant in each randomly picked gene as input and ignore all variant-related scores produced by Exomiser in our evaluation. Exomiser algorithms vary mainly in the background knowledge they use^20^. PhenIX ranks genes solely based on human phenotypes^23^, omitting scores for non-human disease genes. PHIVE integrates input phenotypes with mouse model phenotypes and orthology^22^. ExomeWalker incorporates protein-protein interaction networks^21^, generating a final score via logistic regression.

We utilized the default settings to execute the tools on the generated synthetic datasets from PAVS and ClinVar, inputting the acquired HPO codes for each variant. We assessed the gene scores for each prediction method, excluding variant scores, since our focus is on gene prioritization.

#### Large Language Models used

We used three LLMs as part of this study, GPT-3.5-Turbo^11^ and GPT-4^13^ and the Falcon180B model^24^. GPT-3.5-Turbo is an instruction-following LLM with 20 billion parameters, trained on data up to January 2022. GPT-4 is a multi-modal instruction-following model; the model is commercially available as a black-box model and no technical details are publicly known. We used GPT-4 trained with data up to April 2023. Falcon 180B is an LLM that is publicly available. Falcon 180B was trained on 3.5 trillion tokens primarily consisting of data from the RefinedWeb dataset^24^. We utilized Falcon 180B-Chat, a model with 180 billion parameters, trained up to November 2022. We accessed GPT-3.5-Turbo and GPT-4 via the OpenAI API. Falcon 180B-Chat was run using eight A100 80GB GPUs, following release notes, with the model set to return only high-confidence tokens by setting do_sample to false.

### Prompt engineering

As part of our interaction with the LLMs, we designed structured prompts to engage with the LLMs through their API. We followed the GPT best practice guidelines^25^ to design our prompts. We wrote clear instructions by following the suggested tactics (e.g., ask the model to adopt a persona, use delimiters to clearly indicate distinct parts of the input, specify the output format) and evaluated each prompt on our benchmarking datasets.

We show the experimental prompts in Table 1 and provide examples in the supplements (Supplementary Table S1). We adhere to ethical guidelines to ensure the confidentiality and anonymity of patient information in our interactions with the LLMs. Prompts Q1, Q2, and Q3 are zero-shot^26^, while Q4 constitutes a one-shot, chain-of-thought prompt^26,27^ instructing the LLM specifically on how to perform the gene ranking. In Q1, we instruct the LLM to rank the provided gene list. In Q2, additional patient-related information, including sex and mode of inheritance, is provided. In Q3, the LLM is prompted to rank genes based on their function, expression site, and relevant animal models if there is insufficient information about the gene itself available. Lastly, Q4, a one-shot, chain-of-thought prompt precisely instructs the LLM on the ranking process and the required output format. This prompt provides the LLM with a “reasoning” strategy about how it should identify relevant genes in the presence or absence of different types of information. This chain-of-thought prompt that we designed is inspired by the different types of data that phenotype-based gene- and variant-prioritization methods like Exomiser^20^ use.

**Table 1:** Prompts Crafted.

ID	Type	Prompt
Q1	Zero-shot	A patient presented with these clinical symptoms: [phenotypes]. Rank these genes according to their association with the symptoms of the patient: [genes]
Q2	Zero-shot	A [male/female] patient who is suspected of having a [mode of inheritance/genetic] disease, presented with these clinical symptoms: [phenotypes]. Rank these genes according to their association with the symptoms of the patient:[genes]
Q3	Zero-shot	A [male/female] patient who is suspected of having a [mode of inheritance/genetic] disease, presented with these clinical symptoms: [phenotypes]. Rank these genes according to their association with the symptoms of the patient: [genes]. In the case of insufficient information, still try to rank these genes by using function, site of expression or information from animal models
Q4	One-shot, chain-of-thought	Role: You are an automated ranking system. You take a set of patient signs and symptoms (phenotypes) as input, as well as a set of genes in which a likely pathogenic variant has been identified using a bioinformatics system. You return a ranked list of genes according to the likelihood of the damaging variant in the gene causing the phenotypes of the patient. To do the ranking, first identify if there is any knowledge about mutations in the gene causing the same or similar phenotypes as observed in the patient. Use information about disease and phenotypes, animal models, gene functions, and anatomical site of expression. Automatically rank all genes on the last rank if no evidence exists, and rank all other genes based on the likelihood of causing the phenotypes. Your ranked list should include only the user provided genes and not any other gene. Example: A [male/female] patient who is suspected of having a [mode of inheritance/genetic] disease, presented with these clinical symptoms: [phenotypes]. Rank these genes according to their association with the symptoms of the patient: [genes]. Assistant: “Ranked List:” 1. Gene1 2. Gene2 3. Gene3 ... A [male/female] patient who is suspected of having a [mode of inheritance/genetic] disease, presented with these clinical symptoms: [phenotypes]. Rank these genes according to their association with the symptoms of the patient:[genes]

To identify the prompt to use, we assessed GPT-3.5-turbo’s performance on GPCards using a selection of nine randomly chosen genes and one causative gene retrieved from GPCards. Table 2 shows the performance results of the different queries, including several combinations and variations of the queries. Q2$+$Q3 denotes the utilization of Q3 when Q2 fails, i.e., when the LLM does not rank a given set of genes based on Q2. The symbol Q2−sign indicates substituting “symptoms” with “signs and symptoms”, whereas Q2−pheno represents replacing “symptoms” with “phenotypes” in Q2. Q2-full gene names represents using full gene names instead of their symbols in Q2.

### Analysis of noisy phenotypes

To assess the robustness of our LLM-based approach against noise in gene prioritization, we conducted experiments by systematically altering the phenotypes in patient profiles. For this purpose, we mapped phenotypes from GPCards to HPO using a dictionary of class names and synonyms available in HPO (using HPO version 2019-11-08, following GPCard’s HPO release). For each phenotype, we retrieved its direct parents and direct children in HPO.

To introduce controlled modifications, we applied four different alteration scenarios. (1) Phenotype removal: in this experiment, randomly selected phenotypes were removed from the patient’s profile. (2) Phenotype addition: randomly selected HPO terms that were not already present in the patient’s profile were added to the phenotypes. (3) Phenotype generalization: randomly selected phenotypes from the patient’s profile were replaced with one of their direct parent terms. (4) Phenotype specialization: randomly selected phenotypes of the patient were replaced with one of their direct child terms.

For all scenarios, modifications were applied at predefined proportions (10%, 25%, 50%, or 75%). If a patient had only a single phenotype, no alterations were applied (we identified only one patient profile out of 50 in GPCards with such characteristics). When calculating the number of phenotypes to modify, we used the floor value to determine the number of alterations.

### Rare disease cohort

We applied GPT-4 to 32 families presenting at King Khalid University Hospital (KKUH) in Riyadh. Each family consisted of at least one individual with suspected genetic disease and multiple unaffected family members who provided blood samples at KKUH where DNA was extracted from blood. Using the extracted DNA, we constructed DNA libraries using a QIAGEN QIAseq FX DNA Library kit and sequenced each individual using an Illumina NovaSeq 6000 with an average coverage of 30x for each genome.

We used the bcbio-nextgen toolkit^28^ and standard workflows to align the genomes to the GRCh38 human reference genome, to call variants using the GATK Haplotype caller^29^, and genotype individuals. After variant calling and genotyping, we filtered common variants (minor allele frequency less than 1%) using gnomAD (version 2.1.1)^30^ and the 1,000 genomes project all population frequencies^31^. We then used the suspected mode of inheritance assigned by the clinical geneticist at KKUH based on the observed pattern of inheritance within the family and filtered variants by family pedigree using the SliVar^4^ software. We further removed variants not considered pathologically “impactful” by using the SliVar tool, i.e., excluding synonymous and intronic variants^4^.

We carried out expert assessment of the plausibility of the ChatGPT suggested variants, looking primarily for consistency with existing knowledge and reasonable mechanistic coherence (discussed further in Section 3.3). Our expert assessment of plausibility includes: knowledge of gene function, pathway involvement, experimental findings on whole animal and cellular systems, and phenotypic similarity ( e.g. closely clinically related aspects of developmental delay) amongst other factors. We include reported phenotypes associated with other variants in the same gene, phenotypes caused by members of the same pathway or gene family members, and those that may be reasonably inferred from mechanistic knowledge.

In the light of the expert assessment of the biological and clinical plausibility of candidates, we examined the explanations given by GPT-4 using four criteria, scoring out of 5 for each criterion. Truthfulness; the explanation given using objectively identifiably correct assertions, such as “gene A is a member of gene family X and is involved in Biological process Y”. Informativeness; the explanation gives insight into the causative link between alteration of gene function and phenotype, for example through a previously characterised mechanism. Completeness; the explanation providing a complete rationale for the generation of all the phenotypes seen in the patient as a consequence of the suggested mechanism. Relevance; that the explanation is relevant to the candidate gene and the phenotypes reported and does not, for example, use an explanation that involves a completely different gene or set of phenotypes to those input in the prompt.

### Evaluation procedure

We applied LLMs to the problem of ranking genes based on a set of phenotypes associated with a Mendelian disorder. The input is a set of genes and a set of phenotypes, and the output is a ranked list of genes, with the gene most likely to be causative of the observed phenotypes ranked first. This evaluation reflects the use case where variants are already filtered by different evidence types or machine learning tools predicting pathogenicity, and the remaining variants have to be ranked based on whether the gene products they affect are likely involved in causing the observed set of phenotypes. In this ranking, only a few genes or gene products need to be considered.

We designed prompts in which we asked the LLM to rank a set of genes based on their likelihood of being involved in a set of phenotypes. The phenotypes we used in the prompts are taken from the phenotypes in the datasets, and one gene in the list of genes is the gene associated with the set of phenotypes in the given dataset; the other genes are randomly chosen from all human genes. We evaluated the ranked list of genes generated by the LLMs as output, and determined where the positive gene (from the genotype-to-phenotype information in the datasets) is ranked.

The LLM provides a ranked list of genes, often with explanatory sentences. We disregarded explanations and assessed the LLMs’ performance in ranking the “correct” genes.

The evaluation metrics used in this study are described in Supplementary Materials.

## Results

### LLMs accurately rank candidate genes

We first applied LLMs on a dataset of genotype–phenotype relations derived from GPCards. Initially, we experimented with a set of “zero-shot” and “chain-of-thought prompts”^26^ (see Materials and Methods) by using GPT-3.5 to optimize the prompts.

**Table 2:** Evaluation of GPT-3.5-turbo with various prompts on GPCards.

Hits ($\%$)	Q1	Q2	Q2−sign	Q2−pheno	Q2-full gene names	Q3	Q2$+$Q3	Q4
Hits@1	74	76	76	76	30	62	80	80
Hits@5	92	94	94	94	64	78	98	98
Hits@10	92	94	94	94	100	82	100	100

The numbers indicate the percentage of the causative gene hits at ranks 1, 5, and 10. The notation Q2$+$Q3 denotes the utilization of Q3 when Q2 fails. The symbol Q2−sign indicates substituting “symptoms” with “signs and symptoms”, whereas Q2−pheno represents replacing “symptoms” with “phenotypes” in Q2. Q2-full gene names represent using full gene names instead of their symbols in Q2.

Table 2 presents the results obtained with different prompts using a gene size of 10 on GPCards. Q4 (one shot, chain-of-thought) and Q2+Q3 (zero-shot) outperform other query formats. However, results may not achieve the accuracy 100% at Hits@10 due to hallucinations, such as the causative gene being absent from the LLM list returned. To assess LLMs’ gene ranking performance with an increasing number of discriminated genes, we expanded the random genes added to the causative one from 4 to 99. One-shot chain-of-thought prompting shows potential improvement over zero-shot prompting, with GPT-4 outperforming all tested LLMs (see Table 3 and Supplementary Table S2 for other LLMs).

We repeated the experiment on GPCards five times (see Supplementary Table S3) to determine the stability of the results, and find that the ranking results in the GPCards dataset are stable across multiple repetitions. Across these repetitions, one-shot prompting consistently outperformed other approaches, especially for larger gene lists (50, 75, and 100 genes). Notably, one-shot prompting also showed more reproducible results for gene ranking where we found the mean (M) and standard deviation (SD) for the largest gene set size (100) as M=0.90, SD=0.03 for zero-shot and M=0.94, SD=0.01 for one-shot on GPCards (for full results, refer to Supplementary Table S3).

Furthermore, we investigated the robustness of GPT-4 against noise in the datasets. To this end, we conducted experiments with GPCards, randomly removing, adding and replacing (with their parent or child classes) 10%, 25%, 50%, or 75% of the phenotypes for each patient with 25 genes. Our results showed that GPT-4 remained robust to noise until a significant proportion (75%) of the phenotypes was removed or added, while replacing the phenotypes with their parent or child classes had minimal impact on performance (see Supplementary Table S4).

### LLMs improve on ontology-based ranking methods

While our results on the GPCards dataset show that LLMs can rank genes based on a set of phenotypes specified in natural language, the majority of phenotype-based gene- or variant-prioritization methods rely on input specified in a formal language based on phenotype ontologies^6,20^. The use of an ontology removes ambiguity in phenotype descriptions and enables access to background knowledge contained in phenotype ontologies^9^. To compare the use of LLMs with established ontology-based ranking methods, we followed the same setup in ranking a set of genes and identifying the causative genes given a set of phenotypes, and we compared LLMs with the ontology-based tool Exomiser. Exomiser implements multiple different algorithms for prioritizing candidate genes based on different sources of information; it uses human phenotypes in the PhenIX algorithm^23^, mouse model phenotypes in PHIVE^22^, human and other model organisms phenotypes in hiPHIVE^22^, and protein–protein interaction networks in ExomeWalker^21^.

We used two databases of genotype–phenotype associations for our evaluation and comparison. The first is ClinVar, which is used widely to benchmark variant- and gene-prioritization methods. ClinVar contains associations of variants with diseases (specified using their OMIM identifiers); the OMIM diseases can then be mapped to their phenotypes using the HPO database^7^. For evaluation, we used only variants that have been added after the knowledge cut-off date (2 July 2023 – 7 October 2023) for GPT-4. While ClinVar is a comprehensive dataset of genotype–phenotype relations, it does not associate variants with phenotypes observed clinically but rather with the disorder. Therefore, we also used the PAVS database, a database of phenotype-associated variants in Saudi Arabia, which contains clinically-reported phenotypes and the associated variants. For both sets of variants, we followed a similar procedure as for our previous evaluation: we input the gene affected by the variant together with 4, 24, 49, 74, or 99 randomly chosen genes and asked the LLMs to rank the list of genes given the phenotypes. As GPT-4 was the best-performing model, we only evaluated GPT-4 on this task. Furthermore, since Exomiser outperformed all compared methods, we reported Exomiser’s performance and included all other methods’ performance in the supplementary tables.

Table 3 shows results for ranking genes based on ClinVar variants and phenotypes. GPT-4 with a one-shot chain-of-thought query outperformed a zero-shot query. It ranked genes better than the best performing baseline method (Exomiser, refer to Supplementary Table S5 for other methods) when fewer than 25 genes are included, but its performance decreased compared to Exomiser when ranking more than 25 genes.

**Table 3:** Performance comparison across different Gene Set Sizes and Cohorts.

Size	Metric	GP-Cards		ClinVar			PAVS		
		GPT-4		GPT-4		Exomiser	GPT-4		Exomiser
		Zero shot	One shot	Zero shot	One shot		Zero shot	One shot	
5	Hits@1 (%)	98.00	94.00	93.00	97.00	89.00	93.00	94.80	97.60
	Hits@10 (%)	100	100	100	100	100	100	100	100
	ROC AUC	0.998	0.990	0.991	0.991	0.967	0.989	0.991	0.927
	AUPR	0.985	0.949	0.944	0.972	0.889	0.943	0.956	0.771
25	Hits@1 (%)	78.00	78.00	82.00	85.00	84.00	75.40	77.80	65.60
	Hits@10 (%)	98.00	98.00	94.00	97.00	91.00	97.80	99.00	82.60
	ROC AUC	0.961	0.964	0.964	0.982	0.938	0.970	0.979	0.880
	AUPR	0.770	0.756	0.802	0.856	0.799	0.753	0.784	0.618
50	Hits@1 (%)	76.00	70.00	81.00	87.00	84.00	66.40	68.40	57.20
	Hits@10 (%)	94.00	94.00	96.00	97.00	91.00	91.00	95.20	80.20
	ROC AUC	0.962	0.961	0.971	0.977	0.936	0.949	0.973	0.875
	AUPR	0.723	0.680	0.806	0.786	0.789	0.639	0.677	0.540
75	Hits@1 (%)	62.00	72.00	71.00	74.00	82.00	59.80	61.80	51.60
	Hits@10 (%)	86.00	94.00	82.00	96.00	86.00	83.40	91.40	79.40
	ROC AUC	0.919	0.981	0.925	0.980	0.932	0.992	0.960	0.872
	AUPR	0.576	0.681	0.663	0.742	0.771	0.553	0.595	0.483
100	Hits@1 (%)	60.00	60.00	68.00	70.00	82.00	56.40	57.80	58.60
	Hits@10 (%)	86.00	82.00	81.00	89.00	85.00	77.20	84.80	78.40
	ROC AUC	0.928	0.927	0.911	0.929	0.930	0.895	0.937	0.870
	AUPR	0.343	0.559	0.622	0.668	0.766	0.500	0.539	0.445

Exomiser and the other methods are not applicable to GPCards since the phenotypes are represented in free-text without assigned Human Phenotype Ontology terms.

**Table 4:** Hallucination results.

Model and prompt	GPCards		PAVS		ClinVar	
	Missing	Extra	Missing	Extra	Missing	Extra
GPT-4—zero shot	42.00	00.80	14.48	01.84	15.40	01.60
GPT-4—one shot	14.80	03.60	24.72	03.68	25.00	02.80
GPT-3.5 turbo—zero shot	56.80	00.80	–	–	–	–
GPT-3.5 turbo—one shot	46.40	30.40	–	–	–	–
Falcon 180B—zero shot	44.00	06.80	–	–	–	–
Falcon 180B—one shot	12.00	30.80	–	–	–	–

Missing indicates, the ranked list of genes missing one/more input genes. Extra indicates the ranked list of genes contains one/more genes that are not provided in the input list. The values are percentages obtained by using the results of all the prompts regardless of the gene size (5,25,50,75,100). We have 500, 250, and 2500 prompts for ClinVar, GPCards, and PAVS respectively.

In the case of ClinVar, we used the HPO phenotypes as input for ranking; these phenotypes are identical to the phenotypes associated with the causative gene in the database used by Exomiser, and this may bias the results. To address this, we evaluated genotypes from the PAVS database in Table 3. GPT-4 outperformed all other methods in ranking genes (see Supplementary Table S6 for other baseline methods) in the PAVS dataset, indicating its robustness to noisy phenotype descriptions compared to methods relying on semantic similarity and explicit genotype-to-phenotype databases.

### LLMs reveal candidate genes for undiagnosed cases

We evaluated LLM-based gene ranking using 32 families with undiagnosed genetic diseases (See Supplementary Table S7). All were seen at King Khalid University Hospital (KKUH) in Riyadh, Saudi Arabia. Whole genome sequencing was conducted for affected individuals and family members (see Methods). Data for these families is not publicly available, presenting a challenging “unseen” test case for LLM utility in rare genetic diseases. Variants were filtered by pedigree, mode of inheritance, and allele frequency, retaining rare and potentially impactful variants (see Methods). After filtering, affected individuals had between 1 to 215 candidate variants each (mean 51.90) affecting 1 to 161 genes (mean 37.97).

We used the genes with a potentially impactful variant after all filtering steps as the list of genes to rank, and the phenotypes observed clinically for each family as phenotypes. We used either the zero-shot or single-shot chain-of-thought prompt evaluated earlier. Based on our performance evaluation, we applied only GPT-4 to this cohort.

All 32 families underwent a detailed analysis to assess the biological and clinical plausibility of the top five candidate gene predictions. We assessed whether the top candidate from either the zero or few shot approaches had, in the opinion of two experts, a likelihood or possibility of being the causative gene (see Section 2.4). This assessment, while expert, is inevitably subjective in the absence of direct experimentation, e.g. generating mice or cells carrying the mutant allele, direct 3D molecular structural determination of the mutant protein, or experimental in vitro functional assay. Our criteria broadly overlap with those suggested by Strande at al.^32^ but given the heterogeneity of evidence available across the candidates, only a semi-quantitative approach is possible.

For example, we took into account existing evidence that the gene had already been associated with a closely-related phenotypic description presented in the case in a genotype-to-phenotype database; evidence for loss or gain of function of a gene giving rise to at least two of the phenotypes or phenotype domains seen in the family (for example delay in speech acquisition was regarded as an example of developmental delay and therefore closely related); concordance of gene function or process, as described either by Gene Ontology^33^ or in the literature, with the phenotypic description; phenotypic concordance with loss or gain of function of an ortholog in an experimental model; functional or etiological relationship between the phenotype of the patient and a gene–phenotype (e.g., vermis hypoplasia was regarded as closely associated with seizures/epilepsy or other neurodevelopmental disorders as the two are closely linked). Given the large variation in the severity and spectrum of disease manifestations in many rare diseases^34^, it is doubly important to assess biological plausibility rather than scoring precise and complete phenotype matches.

We evaluated the top five genes for each of the 32 families. All families had ranked candidate genes except one where GPT-4 failed to rank. Of these, 16 cases received at least one gene with a plausibility score of 4 or 5, and 23 genes were deemed plausible by expert evaluation out of a total of 155 scored.

### Explanations, hallucinations, and reproducibility

One of the advantages of LLMs in variant- and gene-prioritization is that they can not only perform the ranking of genes but can also provide explanations for the ranks assigned. However, due to the statistical nature of LLMs, they may also provide output that is factually incorrect, irrelevant, or inconsistent; we collectively refer to these outputs as “hallucinations”.

The first type of hallucination we observed was when the ranked list of genes included genes that were not specified as input, omitted genes that were provided as input, or contained duplicates (see Table 4). All LLMs that we evaluated often omitted genes (up to 56% cases), not ranking all input genes.

Less frequently, LLMs also added new genes to the list to rank. Overall, we found that GPT-4 was more reliable than the other LLMs we evaluated, with the lowest number of hallucinations (both removing or adding genes from the list to rank), and that prompts where phenotypes use structured, ontology-based input instead of free-text were less prone to hallucinations than free-text input (Table 4).

We observed a second type of hallucination in the explanations that LLMs are generated for ranking certain genes. Hallucinations included those that gave an inappropriate response, ones that gave an irrelevant response, and the ones that seemed true and were convincing, but had no basis in fact. The latter poses the most significant concern as it can be challenging to identify and typically requires expert review of available sources and literature.

We manually reviewed the quality of the explanation given by the LLM for its choices of the top five gene candidates for 32 families from our rare disease cohort. In some cases, the LLM gave a single global explanation which was often very general and factually correct but uninformative. In other cases, specific reasons were given for each gene.

We assessed the explanations provided in terms of their truthfulness, informativeness, completeness, and relevance. Truthfulness was assessed by whether the statement was factually true and could be substantiated with facts or reasonable inferences from facts. Informativeness was assessed on how much useful, relevant, or novel information was conveyed by the explanation. Completeness describes whether the explanation provides a rationale for all aspects of the patient phenotypes. Relevance was assessed by the degree to which the explanation for gene association was biologically or clinically relevant to the phenotypes. A summary of results is available as Supplementary Table S7. We show that most explanations were factually correct (Truthfulness: mean: 3.8, SD: 1.9), although usually uninformative (Informativeness: mean: 2.2, SD: 1.6) and incomplete (Completeness: mean 1.8, SD: 1.4), and often not relevant to the phenotypes observed (Relevance: mean 2.1, SD: 1.8). We also ranked plausibility for the gene being causative of the phenotypes; overall, across the top five genes, plausibility had a mean of 1.6 (SD: 1.7). However, if we only consider the highest-scoring gene among the five genes we evaluated, the mean plausibility is 3.7 (SD: 1.2), and for 15 families out of 32, we identified at least one candidate scoring with a plausibility of 4 or 5.

While most explanations were truthful, there are some where we have been unable to identify evidence for the truth of the statements made. For example, in one family where the affected individual has a wide range of phenotypes, GPR107 is the second-ranked candidate gene and we are provided with the following explanation: “GPR107: This gene encodes a protein that is a member of the G protein-coupled receptor superfamily. This protein has been shown to be a receptor for glucose, and is widely expressed in the central nervous system. While not directly linked to the symptoms, it could potentially be involved due to its role in glucose metabolism.” It is true that GPR107 is a *G* protein-coupled receptor, and it may be involved in glucose metabolism through its action on glucagon physiology via its binding to neuronostatin^35^. However, we have been unable to find any evidence that it binds glucose. Therefore, while the overall assertion is largely true, the LLM has hallucinated part of its explanation: GPR107 does not bind glucose. Furthermore, while there is a wide range of phenotypes reported, there is no clear common linkage to glucose metabolism and we consider this to be another type of hallucination, i.e., generation of an irrelevant response.

We observed a similar hallucination in another family where the affected individual has astigmatism, Legg-Perthes^36^, intellectual disability, and short stature. The explanation given for the top suggestion (SMPD3) is: “This gene is associated with Legg-Perthes disease, a condition that affects the hip joint in children and can lead to short stature.” While it is true that, in principle, a hip disorder can lead to short stature, there is no discoverable link between SMPD3 and Legg-Perthes disease, and the disorder is primarily associated with Col2A1 (OMIM:150600  ^37^ ). It is, however, true that loss of function of the mouse ortholog gives rise to disproportionate dwarfism^38^, which is in principle a match. However, there are no behavioural or ocular phenotypes in these mice.The LLM has made a partial connection with the gene and the phenotypes, but the assertion that it is known to be involved in Legg-Perthes disease is hallucinatory. In a third type of hallucination, GPT-4 invented a syndrome, “TOR3A syndrome” with associated phenotypes bearing some relation to those of the patient. We can find no reference in the literature to “TOR3A syndrome” but TOR1A is associated with torsion dystonia^39^; OMIM: 128100. GPT-4 seems to have associated the phenotype of one gene with another closely related in name and function. We have seen this problem several times. For example, MYH4 and MYH14, where MYH14 is associated with autosomal deafness (OMIM: 600652) which is part of the patient phenotype, but MYH4, the given candidate, has no association with deafness that we can discover.

### Comparison of GPT-4 and Exomiser results on KKUH families

We compared the performance of GPT-4 and Exomiser in gene prioritization across 32 families, focusing on the top 5 candidate genes when available from each tool. Supplementary Table S8 presents the integrated analysis, including plausibility scores for the top five genes suggested by Exomiser and GPT-4. Overlapping genes between the two methods are highlighted for clarity. Additionally, Supplementary Table S9 provides a comparative summary of the performance of GPT-4 and Exomiser across all families. In total, we analyzed 142 and 156 genes from GPT-4 and Exomiser respectively. Among these top rankings, 42 genes (16.27% of the combined predictions) were suggested by both tools.

When evaluating gene plausibility, GPT-4 identified 23 plausible genes across 16 families, while Exomiser identified 18 plausible genes across 11 families, with 10 families overlapping between the two methods. Of these 10 families, seven had overlapping genes predicted by both tools, with a total of 10 overlapping plausible genes identified (32.26%): three families had two overlapping plausible genes each, and four families had one overlapping plausible gene. The remaining seven families each had a single plausible gene identified by one of the tools: six by GPT-4 and one by Exomiser. We further compared the tools based on the five solved cases where the causative genes were known. GPT-4 demonstrated superior performance by identifying all five causative genes (100% success rate) within its top 5 predictions. In contrast, Exomiser identified two of the five causative genes (40% success rate) in its top 5 predictions.

Our comparative analysis reveals that while both tools can effectively prioritize candidate genes, GPT-4 showed higher accuracy in ranking both plausible and causative genes within its top 5 predictions. The modest overlap in predictions (16.27% overall, 32.26% in plausible genes) suggests they employ different approaches to gene prioritization, potentially making them complementary in clinical genetic analysis.

## Discussion

We studied the use of LLMs for the task of gene prioritization based on phenotypes, a task that has traditionally been thought to rely on structured background knowledge. Our results demonstrate that LLMs can perform as well or better than custom-built tools for this task. Notably, our time-based ClinVar experiment revealed that the lack of provenance and original training data has no impact on LLM performance (See Supplementary Table S5). Phenotype-based methods for ranking candidate genes consist of two main components: a knowledge-base of genotype-to-phenotype relations, and a similarity measure^6^. Often, they also contain structured background knowledge about how phenotypes are related, usually in the form on a phenotype ontology^9^. They also use a similarity measure based on the background knowledge, thereby making it a semantic similarity measure^8^. To perform better than phenotype-based gene prioritization methods, LLMs need to be able to replace these two main components. LLMs obtain their background knowledge from information covering structured datasets, ontologies, and literature; the content of genotype-to-phenotype databases will therefore be included at least to large parts as training data or extracted from the literature (for example in the form of clinical case report^40^). LLMs also seem to be able to compute similarity between phenotypes as well or better than the custom-built similarity measures in Exomiser, demonstrated in particular when using clinical phenotype descriptions as input to the ranking model (Table 3).

LLMs can accept various types of input, including arbitrary text, as demonstrated in one dataset not reliant on the HPO vocabulary. While Exomiser’s baseline methods use HPO codes, we solely employed natural language labels for LLM input in our experiments. Although the biomedical community has created valuable phenotype ontologies like the HPO to aid gene prioritization, our findings suggest that structured phenotypes may not be essential and could even hinder success. This could be due to incomplete ontology information leading to errors or limitations in expressing information compared to natural language input for LLMs. While our observations focus on one aspect of phenotype ontologies, future considerations should weigh the benefits of ontologies against the capabilities of advanced LLMs for various tasks.

Our experiments also show that LLMs go beyond gene prioritization systems in that they can provide explanations for their results. Furthermore, LLMs also have the potential to refine and update ranking results interactively.

The best use of LLMs may therefore be not as a simply ranking system but rather as an interactive diagnostic assistant. We also attempted to estimate how robust LLMs are to noise, and find that they tolerate different types of noise, including omitting phenotypes, adding unrelated phenotypes, and using more generic or more specialized phenotypes. Using the GPCards dataset, we also evaluated how stable results are when queries are repeated, and find that results generally remain the same with only small differences in repeated queries. However, for both the ClinVar and PAVS datasets, our analysis is based on single queries, i.e., we did not repeat the queries with different random gene sets, due to the larger size of the datasets compared to GPCards. We therefore cannot determine how stable the results are when performed multiple times. Furthermore, future work still needs to address “hallucinations” as well as ways to quantify uncertainty; knowledge graphs and ontologies may provide ways to solve the problem of hallucination^41,42^ by providing structured knowledge.

One potential limitation of our study is that the language models we assessed were trained on text (i.e. literature articles) containing variants found in our testing set. This issue also extends to the Exomiser tool, which relies on reported gene phenotypes. To address this, we only evaluated variants post the GPT-4 cut-off date. For future studies, a prospective approach utilizing language models for genetic disease diagnosis should be considered. This study could explore other types of genomic variants, such as non-coding and structural variants, along with methods for incorporating structured background knowledge to enhance learning.

## Supplementary Information

Supplementary Information.

## Data Availability

Primary or derived data from the families that were sequenced and analyzed is available only for researchers with access approved by the responsible IRB. Any requests for data access should be addressed to the Institutional Bioethics Committee at King Abdullah University of Science and Technology and the Institutional Review Board (IRB) at King Saud University. All other data and code used in this study are available at https://github.com/bio-ontology-research-group/LLM_GenePrioritization.

## Declarations

### Compliance with guidelines

All methods were carried out per the guidelines and regulations laid out by the institutional bioethics committees, the Declaration of Helsinki, and applicable laws and regulations governing research involving human subjects.
